# 4′-*O*-methylpyridoxine: Preparation from *Ginkgo biloba* Seeds and Cytotoxicity in GES-1 Cells

**DOI:** 10.3390/toxins13020095

**Published:** 2021-01-26

**Authors:** Jin-Peng Zhu, Hao Gong, Cai-E Wu, Gong-Jian Fan, Ting-Ting Li, Jia-Hong Wang

**Affiliations:** 1 College of Light Industry and Food Engineering, Nanjing Forestry University, Nanjing 210037, Jiangsu, China; zjp@njfu.edu.cn (J.-P.Z.); fangongjian@njfu.edu.cn (G.-J.F.); Litingting@njfu.edu.cn (T.-T.L.); njfuwjh@njfu.edu.cn (J.-H.W.); 2 College of Food Engineering, Xuzhou University of Technology, Xuzhou 221018, Jiangsu, China; 11902@xzit.edu.cn; 3 Co-Innovation Center of Efficient Processing and Utilization of Forest Resources, Nanjing Forestry University, Nanjing 210037, Jiangsu, China

**Keywords:** *Ginkgo biloba* seeds, 4′-*O*-methylpyridoxine, GES-1 cells, apoptosis, extraction

## Abstract

*Ginkgo biloba* seeds are wildly used in the food and medicine industry. It has been found that 4′-*O*-methylpyridoxine (MPN) is responsible for the poisoning caused by *G. biloba* seeds. The objective of this study was to explore and optimize the extraction method of MPN from *G. biloba* seeds, and investigate its toxic effect on human gastric epithelial cells (GES-1) and the potential related mechanisms. The results showed that the extraction amount of MPN was 1.933 μg/mg, when extracted at 40 °C for 100 min, with the solid–liquid ratio at 1:10. MPN inhibited the proliferation of GES-1 cells, for which the inhibition rate was 38.27% when the concentration of MPN was 100 μM, and the IC_50_ value was 127.80 μM; meanwhile, the cell cycle was arrested in G2 phase. High concentration of MPN (100 μM) had significant effects on the nucleus of GES-1 cells, and the proportion of apoptotic cells reached 43.80%. Furthermore, the Western blotting analysis showed that MPN could reduce mitochondrial membrane potential by increasing the expression levels of apoptotic proteins Caspase 8 and Bax in GES-1 cells. In conclusion, MPN may induce apoptosis in GES-1 cells, which leads to toxicity in the human body.

## 1. Introduction

*Ginkgo biloba* L. has existed on the earth for more than 200 million years since the Carboniferous period and has been known as a “living fossil” in China [1]. The seeds of *G. biloba* have been used as food and traditional medicine to treat coughs, asthma, and urinary diseases, which was recorded in “Compendium of Materia Medica” [2]. According to previous reports, overconsumption of *G. biloba* seeds can lead to toxicosis and even death [1]. Wada et al. revealed that 4′-*O*-methylpyridoxine (MPN), a derivative of vitamin B_6_, is responsible for the poisoning caused by the overconsumption of *G. biloba* seeds [3]. Scott et al. had found that 4′-*O*-methylpyridoxine-5′-glucoside (MPNG), the glucoside of MPN, is also toxic [4].

In previous reports, distilled water was used to extract MPN analogs from *G. biloba* seeds, and these analogs were analyzed using high-performance liquid chromatography (HPLC) [5]. Moreover, different temperatures (room temperature, 40 °C, 70 °C, and 100 °C) were used in sample preparation [6,7,8,9]. However, MPN analogs, which will convert into MPNG, were unstable when the *G. biloba* seeds were heated [10]. Given the differences in toxicity between MPN and MPNG, determining the native distribution of MPN analogs in *G. biloba* seeds is important. Meanwhile, vitamin B_6_ contents can be influenced by physical and chemical factors, such as heat, light, and pH [11]. Although MPN analogs are structurally similar to vitamin B_6_, little information is known about the effects of extract conditions on MPN analogs during sample preparation. Hence, sample preparation procedures must be optimized to investigate the characteristics of MPN.

According to previous research, MPN has a certain toxic effect on the body’s nervous system, gastrointestinal system, and respiratory system both with in vivo and in vitro experiments [12]. Miwa et al. have found that excessive consumption of *G. biloba* seeds caused frequent vomiting and generalized convulsions [13]. Kajiyama et al. have reported that a young girl presented with vomiting and diarrhea after eating *G. biloba* seeds, meanwhile the concentration of MPN had raised to a high level in her serum [14]. However, the toxic effects of MPN on humans at the cellular level have yet to be investigated.

Apoptosis, or programmed cell death, refers to a form of cell death in an active and inherently controlled manner that eliminates cells that are no longer wanted [15]. In addition to maintaining cell stability, the process of apoptosis also occurs under the induction of diseases such as tumors, diabetes, radiation, and drugs [16,17,18]. Cell and nuclear shrinkage, chromatin condensation, formation of apoptotic bodies and phagocytosis by neighboring cells characterize the main morphological changes of the apoptosis process [16]. Besides, the process of apoptosis is controlled by multiple genes, which mainly include *Bcl*-2 family, Caspase family, oncogenes such as C-myc and tumor suppressor gene P53, and the disorder of apoptosis process is often accompanied by abnormal expression of these genes [19].

This work aimed to explore and optimize the extraction method of MPN from *G. biloba* seeds. Furthermore, the toxicity of MPN was investigated at the cellular level by administration in human gastric epithelial (GES-1) cells. The mechanism of MPN inhibitory and apoptosis on GES-1 cells provides a reference for the in-depth study of the cytotoxicity of MPN compounds in *G. biloba* seeds.

## 2. Results

### 2.1. Optimization of MPN Extraction Conditions by Orthogonal Test

**Figure 1:**
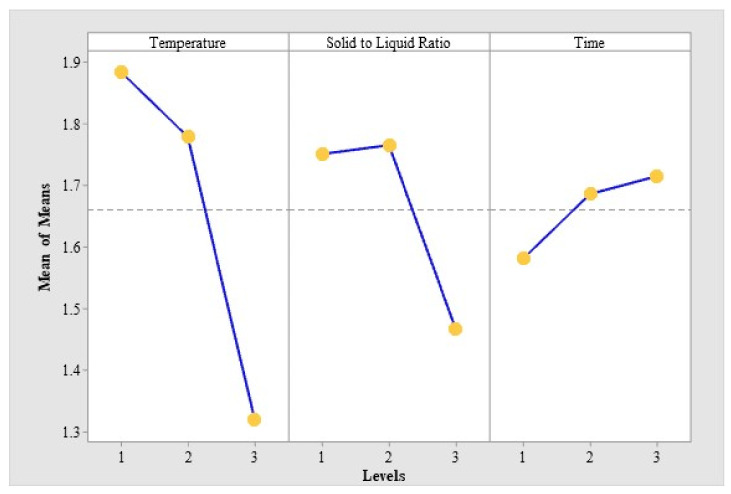
The average value of per level for each factor.

Based on the L_9_(3^4^) orthogonal test, results were calculated and showed in Table 1. In order to facilitate comparative analysis, Figure 1 showed the average value of each factor at each level for different test results. As can be seen in Figure 1, the best combination of theoretical process parameters is A_1_B_2_C_3_.

The temperature (A), as the most influential factor, has a dominant effect on the extraction amount of MPN (Table 2). The verification test was carried out between A_1_B_2_C_3_ and A_1_B_2_C_2_, as the experimental combination of the best results in Table 1 is A_1_B_2_C_2_. Finally, the optimum extract conditions of MPN were the temperature of 40 °C, the solid–liquid ratio of 1:10 and the time of 100 min, in which the extraction amount of MPN can reach 1.933 μg/mg, according to the results of the verification test.

### 2.2. Inhibition of MPN at Different Concentrations on GES-1 Cells Activity

**Figure 2:**
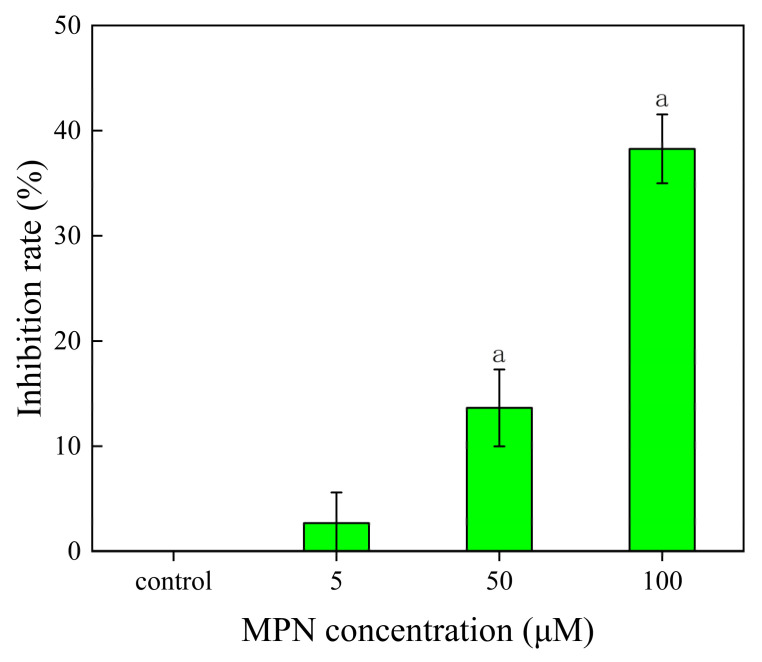
Inhibition of MPN on human gastric epithelial (GES-1) cells; “a” represents the difference between different MPN concentrations compared with the control group at *p* < 0.05.

MPN had no inhibitory effect on GES-1 cells when the concentration was 5 μM compared with the control group (Figure 2). The MPN had significant inhibitory effects on GES-1 cells at 50 μM and 100 μM, with inhibition rates of 13.64% and 38.27%, respectively. The IC_50_ value of MPN to GES-1 cells was 127.80 μM, which was calculated by GraphPad Prism.

### 2.3. Hoechst 33342 Staining Assay 

**Figure 3:**
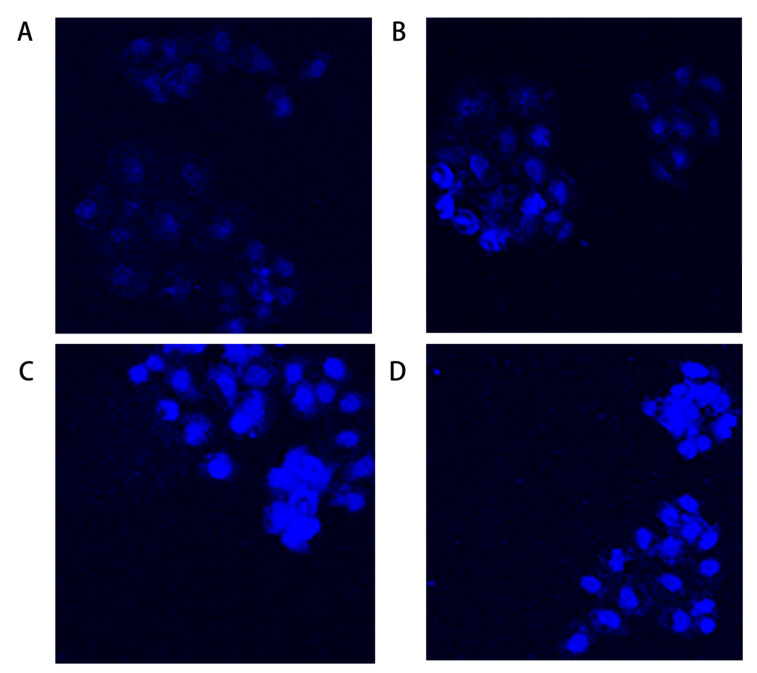
The GES-1 cells were treated with different concentrations of MPN and Hoechst 33342 stain was used to observe the alterations in the morphology of cell nuclei. There was a considerable change in the morphology of the cell nuclei as compared to the control group. Untreated control GES-1 cells (**A**) and 5 μM group (**B**) showed no sign of apoptosis after staining with Hoechst 33342. The fluorescent nuclei showed obvious aggregation after being treated with 50 μM of MPN (**C**), and especially in the 100 μM group (**D**).

In order to observe the morphological alterations of the nucleus and the cytoplasm, GES-1 cells were treated with different concentrations of MPN and subsequently stained with fluorescent stains like Hoechst 33342. Most GES-1 cells in the blank control group and the 5 μM treatment group showed uniform light blue fluorescence (Figure 3). It revealed that the morphology of the GES-1 nucleus was completely round or oval, evenly distributed, and without obvious apoptosis. As the concentration of MPN increased, the dense fluorescence appeared, and in apoptotic cells’ nuclei, the fluorescence is brighter. The results indicated that a high concentration of MPN can have an effect on the fluorescence intensity of GES-1 cells’ nuclei after staining.

### 2.4. Induction of Apoptosis of GES-1 Cells by Different Concentrations of MPN 

**Figure 4:**
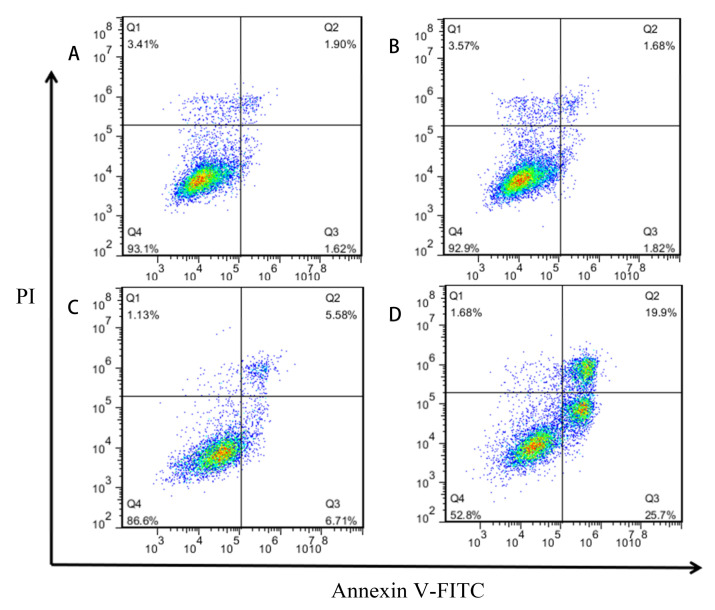
Effects of MPN on apoptosis in GES-1 cells. Cells were collected after the treatment with different concentration of MPN, (**A**) blank control, (**B**) 5 μM, (**C**) 50 μM, (**D**) 100 μM, for 16 h; apoptosis was evaluated using Annexin V-FITC/PI double staining followed by flow cytometry analysis. Both abscissa and ordinate represent the relative fluorescence intensity. Percentage of living cells (Q4), early apoptosis cells (Q3), and late apoptosis cells (Q2) showed in the flow cytometry chart.

As shown in Figure 4, most of the cells in the blank group and the 5 μM treatment group were in the lower left quadrant. The survival rates of the two groups of cells in this area were calculated as 92.90% and 90.80%, respectively (Table S4). There is no significant difference in the proportion of apoptotic cells, which appeared at upper right and lower right quadrants. With the increase in MPN concentration, the proportion of living cells (lower left quadrant) decreased significantly. And early apoptotic cells (lower right quadrant) increased significantly, while late apoptosis and necrotic cells (upper right and upper left quadrant) also gradually increased. It can be found that the apoptosis of GES-1 cells is related to the concentration of MPN. When the concentration of MPN was 100 μM, the apoptosis rate of GES-1 cells was the highest (43.80%).

### 2.5. Effects of MPN at Different Concentrations on GES-1 Cells Cycle

**Figure 5:**
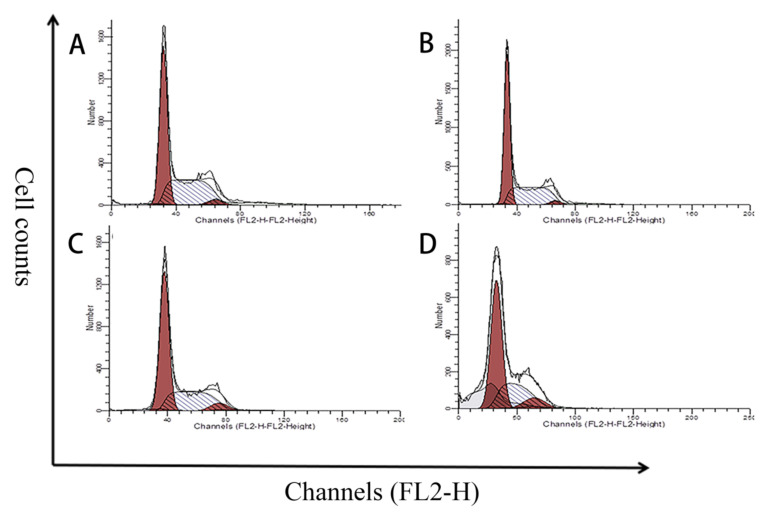
Flow cytometric histograms for the effect of MPN on the cell-cycle distribution of GES-1 cells. The abscissa represents the relative fluorescence intensity, while the ordinate represents the number of cells. The two red peaks indicate that the cells are in the G1/G0 and G2/M phases, while the middle peak indicates that the cells are in the S phase. The GES-1 cells lines were (**A**) treated with medium for a control, (**B**) treated with 5 μM of MPN, (**C**) treated with 50 μM of MPN, and (**D**) treated with 100 μM of MPN. An increase in G1/G0 and G2/M, while a decrease in S was observed in (D) for GES-1 cell cycle treated with 100 μM of MPN.

When the concentration of MPN was 5 and 50 μM, MPN had no significant effect on the ratio of cells in G1/G0 and S phases (Figure 5). When the administration concentration of MPN was 100 μM, the number of GES-1 cells in the G1 and G0 phases increased significantly, while the percentage of cells in the S phase decreased significantly. For the G2/M period, there was no significant change between 5 μM group and the blank group. It can be seen in Table S5, as the concentration of MPN increased, the ratio of G1/G0 and G2/M phases increased significantly, and the percentage of S phase cells decreased. The cell cycle of GES-1 cells has been blocked in S phase, which led cells to be prevented from undergoing a normal cell proliferation cycle, finally inducing apoptosis.

### 2.6. Effects of MPN at Different Concentrations on the Mitochondrial Membrane Potential of GES-1 Cells

Mitochondrial membrane potential (ΔΨm) decreases early in apoptosis. When the mitochondrial membrane potential is at a high level, JC-1 (5,5′,6,6′-tetrachloro-1,1′,3,3′-tetramethylbenzimidazolylcarbocyanineiodide) accumulates in the mitochondrial matrix to form a red fluorescent polymer. When the mitochondrial membrane potential at a low level, JC-1 is a monomer and produces green fluorescence. Therefore, changes in cell fluorescence color were detected before and after administration, which can determine changes in mitochondrial membrane potential.

**Figure 6:**
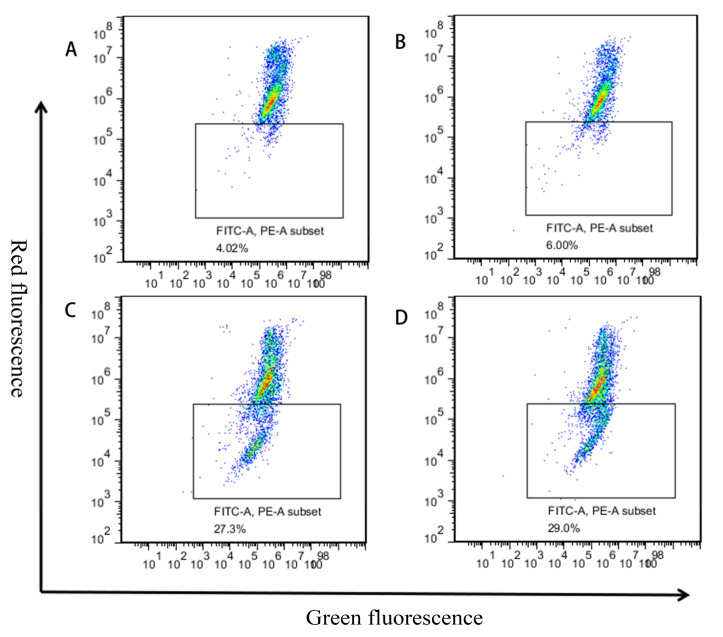
Effect of MPN on the mitochondrial membrane depolarization (JC1) in GES-1 cells. The GES-1 cells were treated with MPN for 16 h. (**A**) Blank control, (**B**) 5 μM, (**C**) 50 μM, (**D**) 100 μM. JC-1 staining for Δψm, observed by flow cytometry. Both abscissa and ordinate represent the relative fluorescence intensity.

JC-1 is aggregated and GES-1 cell mitochondrial membrane potential is high in the blank group and the 5 μM group (Figure 6). Meanwhile, ΔΨm is normal and there is no significant change in green fluorescence intensity. With the increase in MPN concentration, JC-1 is dispersed, and the green fluorescence intensity increases accordingly. When the treatment concentration is 100 μM, the green fluorescence intensity reaches 30.35%, which is a maximum value (Table S6).

### 2.7. Effects of MPN at Different Concentrations on the Expression of Caspase 8 and Bax in GES-1 Cells

The caspase-dependent apoptotic pathway is main characterized by Caspases and *Bcl*-2 family, which upon activation will trigger cell apoptosis. After treated with different concentrations of MPN, the expression of Caspase 8 and Bax in GES-1 cells were detected by Western blotting.

**Figure 7:**
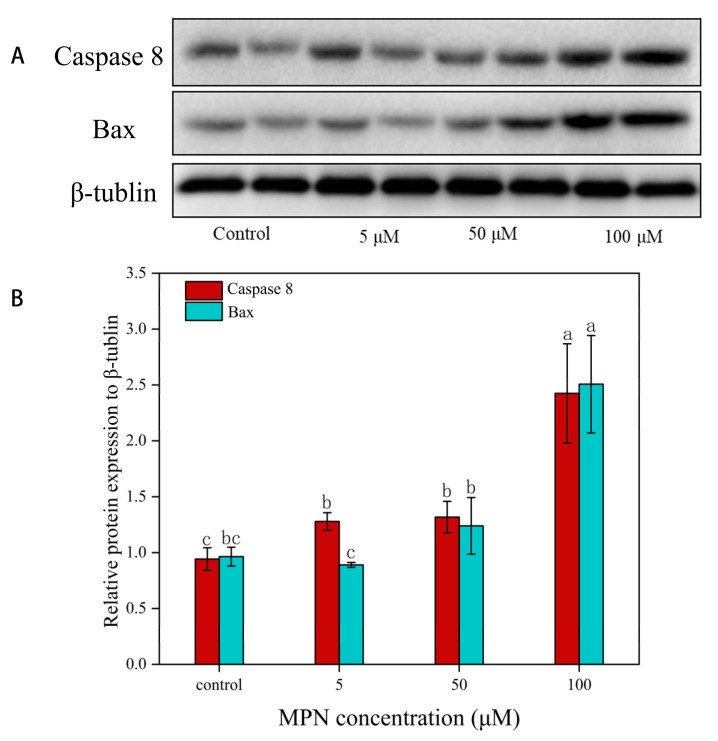
Effect of MPN on the expression of Caspase 8 and Bax in GES-1 cells by Western blot. In the Western blotting analysis, cell lysates were subjected to SDS-PAGE, with β-tublin used as an internal control. Signals of proteins were visualized with an ECL detection system. The results were representative of three independent experiments. **(A**) Western blotting analysis for detecting Caspase 8 and Bax protein levels after indicated treatment; (**B**) relative protein expression level of Caspase 8 and Bax to β-tublin. Different letters (a–c) indicate the difference of different administered MPN concentrations at *p* < 0.05.

As an internal reference, the expression of β-tublin did not correlate with the concentration of MPN. With the increase in MPN concentration, the expression level of Caspase 8 in GES-1 cells was increased gradually compared with the internal reference, and its expression level depended on the concentration of MPN (Figure 7). The expression of Caspase 8 reached the highest level when the MPN concentration was 100 μM. There was no significant difference in the expression level of Bax between the blank and the 5 μM group. When the concentration of MPN continued to increase, the expression level of Bax also increased gradually. These results revealed that the expression levels of Caspase 8 and Bax in GES-1 cells can be increased by MPN, which can also activate the apoptosis pathway in the cell.

### 2.8. Correlation Analysis

The correlation matrix presented in Table S7 shows that a certain degree of correlation existed between the various indicators. For example, inhibition rate and apoptotic rate were positively correlated (r^2^ = 0.994, *p* < 0.01). Apoptotic rate and the number of S phase cells were negatively correlated (r^2^ = −0.997, *p* < 0.01). It confirms our previous conclusion that a high dose of MPN can block cell cycle in S phase. There was also a positive correlation between apoptosis rate and the expression of Caspase 8 and Bax in GES-1 cells, for which r^2^ was 0.984 (*p* < 0.05) and 0.995 (*p* < 0.05), respectively.

## 3. Discussion

This study is the first to investigate the toxicity of MPN on humans at the cellular level. MPN had no significant inhibitory effect on GES-1 cells when the concentration was 5 μM. While, when the concentration of MPN was 100 μM, the inhibition rate to GES-1 cells reached 38.27%. Meanwhile, the IC_50_ value of the GES-1 cells was 127.80 μM. Patients with ginkgo poisoning will exhibit gastrointestinal symptoms such as nausea, vomiting, abdominal pain, and diarrhea, while respiratory symptoms are less common [12]. According to some in vitro studies, MPN showed strong toxicity to nerve cells, gastric mucosal epithelial cells, and small intestinal epithelial cells [12]. These results indicated that the toxicity of MPN has dose-dependence and specificity.

Pyridoxine (PN) is one of the interconvertible pyridine compounds of vitamin B_6_. Studies have shown that overconsumption of PN can cause human poisoning [20,21]. Fiehe et al. found that MPN can be synthesized by PN in the presence of S-adenosyl methionine [22]. When the MPN administration concentration was 50 and 100 μM, it caused apoptosis of GES-1 cells. Hoechst 33342 staining found that high-dose MPN treatment can change the shape of the nucleus of GES-1 cells. Cell cycle detection further found that when the concentration of MPN was 100 μM, the proportion of cells in G1/G0 and G2/M phases increased significantly, while the proportion of cells in S phase decreased significantly. These results show that MPN may cause disturbance of DNA metabolism in GES-1 cells. Lorenzo et al. reported that the addition of PN can induce DNA damage in non-small cell lung cancer cells, aggravating the apoptosis of cancer cells [23]. In addition, this study also found that GES-1 cells showed obvious apoptosis after MPN administration. When concentration of administered MPN increased from 50 to 100 μM, the proportion of apoptotic cells increased from 14.46% to 43.80%.

The cell apoptosis has two different pathways that include an extrinsic pathway and an intrinsic pathway. Mitochondria serve a pivotal role in the intrinsic pathway and are involved in drug-induced apoptosis; in addition, members of the *Bcl*-2 family take part in intrinsic pathway regulation, including regulation of the Bax gene [24]. Caspases are the core of apoptosis mechanism, as they act as both the promoters and the executors of cell death [25]. Furthermore, caspase 8 is a key enzyme acting in the upstream apoptosis pathway, and also acts as an important apoptosis initiation factor by activating almost all of the downstream caspases in the apoptotic cascade [26]. *Bcl*-2 family proteins can mediate the activation of cysteine aspartic acid-specific proteases (caspases) for inducing apoptosis [27,28]. Bax, a pro-apoptosis *Bcl*-2 family member, translocates from the cytoplasm to the mitochondria in response to stimulation, which subsequently results in the release of cytochrome *c* and cell apoptosis [29,30].

The mitochondrial membrane potential will be formed if the asymmetric distribution of protons and other ion concentrations occurs on both sides of the inner membrane, as the energy generated by mitochondria is stored in the inner mitochondrial membrane [31]. The mitochondrial membrane potential is more sensitive to apoptosis, and changes in membrane potential can be detected early in apoptosis [32]. It was found that the mitochondrial membrane potential of GES-1 cells was reduced significantly when the concentration of MPN was 50 and 100 μM. In addition, when the mitochondrial membrane was depolarized cytochrome *c* was also released [33]. Cytochrome *c* can combine apoptotic protease activating factor-1 and pro-caspase-9, which form apoptosomes, activating caspase 9 and caspase 3, which inhibit cells via the mitochondrial-mediated pathway [34]. In previous reports, the increased level of cytochrome *c* and caspase 8, which can stimulate activation of caspase 3, has been described as a key component in the execution stage of apoptosis inducing final apoptosis [35]. After MPN treatment, the expression level of the two proteins in GES-1 cells increased significantly. Previous studies also reported that PN can induce apoptosis of SHSY5Y cells, alongside the increased expression level of Caspase 8 and Bax, which further indicated that MPN also may induce apoptosis in GES-1 cells [36]. However, a more accurate mechanism of MPN-induced GES-1 apoptosis demands further in-depth study.

VB_6_ has important biological functions including anti-immune response, anti-tumor and antioxidant activities [37,38]. Plant-based food is an important source for human to obtain VB_6_, as only plants and microorganisms can synthesize VB6 [39]. According to previous studies, the content of VB_6_ in *Ginkgo biloba* seeds is 400.44–586.82 μg/g, which was higher than that of common foods (e.g., milk, wheat and potato) [40,41]. Some scholars speculated that MPN in Ginkgo seeds may act as an antagonist of VB_6_ in the human body, for that MPN is similar to the PN form of VB_6_ [42]. It is well known that VB_6_ is also involved in protein synthesis and catabolism, amino acid metabolism, synthesis of certain nerve mediators, nucleic acid, and DNA metabolism in the human body [43]. In vitro studies have shown that overconsumption of PN can interfere with the VB_6_ salvage pathway in the body or directly inhibit the activity of amino acid metabolizing enzymes such as tyrosine decarboxylase and alanine aminotransferase [32]. Related studies have shown that the intake of MPN can also cause disturbances in the VB_6_ salvage pathway in rats, such as an increase in pyridoxal (PL) content and a decrease in pyridoxal-5′-phosphate (PLP) content [44]. On the other hand, MPN can be phosphorylated by PL kinase to form 4′-*O*-methylpyridoxine-5′-phosphate (MPNP), and then it can compete with pyridoxal-5′-phosphate (PLP) as a coenzyme of glutamate decarboxylase to inhibit its activity [45]. Combined with the results of this study, it can be speculated that MPN may inhibit cell growth and induce apoptosis through the following two aspects. Firstly, MPN interfered with the VB_6_ salvage pathway, affecting the normal metabolism of intracellular substances such as amino acid metabolism, protein synthesis, and DNA replication. Secondly, MPN, as a toxic factor, induced apoptosis in GES-1 cells directly.

## 4. Conclusions

In summary, this study explored and optimized the extraction method of MPN from *G. biloba* seeds by orthogonal testing and is the first study to demonstrate the toxicity of MPN on humans at the cellular level. The findings of this study suggested that MPN initiated apoptosis of GES-1 cells in a dose-dependent manner. Through this process, MPN with high concentration can significantly increase the expression levels of Caspase 8 and Bax in GES-1 cells, while reducing the mitochondrial membrane potential, which may lead the release of cytochrome *c* and stimulate activation of caspase 3, a downstream caspase. These novel observations further support the hypothesis that MPN can induce apoptosis in GES-1 cells, doing harm to human health. It is of great value to study the cytotoxicity of MPN compounds in *Ginkgo biloba* seeds.

## 5. Materials and Methods

### 5.1. Sample Preparation

*G. biloba* seeds were harvested in Taizhou (China) in 2019. Fresh seeds were shelled and then freeze-dried (500 g) for two days. Afterward, the samples were ground and stored at −80 °C until analysis. The remaining fresh seeds were stored at 0 °C until use.

### 5.2. Optimization of MPN Water Extraction Process

Based on single-factor experiments, the impact of temperature, time, and solid-to-liquid ratio on extraction rate was discussed according to orthogonal testing. According to Figure S3, freeze-dried powders of *G. biloba* seeds (400 mg) were suspended in ultrapure water with different solid-to-liquid ratios (1:5, 1:10, 1:15). From Figures S1 and S2, the resulting solution was continuously shocked at different temperatures (40 °C, 50 °C, 60 °C) for different times (60 min, 80 min, 100 min) at 220 rpm (Jinghong, model THZ320, Shanghai, China). Afterward, the solution was centrifuged at 10,000 rpm for 25 min at 4 °C (Sigma, Model 2-16K, San Francisco, CA, USA), and the supernatant was filtered through a 0.45 μm syringe filter (Jinglong, Tianjing, China). The extraction of MPN was injected into the HPLC system, for which the conditions and steps were presented in our previous works [46]. The validation of HPLC method, which contained preparation of standard curve, recovery test, and precision test, was conducted according to our previous works [47].

### 5.3. Cell Culture

Human gastric mucosal epithelial cells (GES-1) were purchased from Enogene Biotechnology Company (Nanjing, China). The cells were maintained Dulbecco’s Modified Eagle Medium (DMEM) (Gibco Company Inc., Grand Island, NE, USA) supplemented with 10% Fetal Bovine Serum (FBS) (ScienCell Research Laboratories Company Inc., Carlsbad, CA, USA) in a humidified incubator with 5% CO_2_ and 37 °C (MCO-15AC, Sanyo, Osaka, Japan) for 24 h. Moreover, the inhibitory effect of MPN on cells under different concentrations and different treatment times (6, 10, 16, 24, 48 and 72 h) have been studied, in previous studies. According to Figure S4, when the concentration of MPN exceeded 50 μM, the inhibition rate of MPN to GES-1 cells was higher both after 16 h and 24 h than others. Furthermore, as the IC_50_ value of 16 h treatment time was lower than others (Table S8), 16 h of treatment time was chosen for further studies.

### 5.4. Cell Viability Assay

Cell viability was examined by using Cell Counting Kit-8 (CCK-8) (E1CK-000208-10, Enogene Biotechnology Co., Nanjing, China) according to the manufacturer’s instructions. Briefly, GES-1 cells were exposed to different concentrations of MPN (0, 5, 50, and 100 μM). After 16 h of incubation, 10 µL of CCK-8 solution was added to the wells and incubated for additional 4 h at 37 °C. Finally, the absorbance was detected at 450 nm by a microplate reader (MUTISKAN-MK3, Thermo scientific, Waltham, MA, USA). The results were expressed as percentages of cell viability.

### 5.5. Hoechst 33342 Stain

MPN was added to GES-1 cells, after which the cells were collected and inoculated in a 96-well plate. After 16 h of treatment, the cells were washed with phosphate buffered saline (PBS) twice, and then incubated with 1 μg/mL Hoechst 33342 Stain (Beyotime Biotechnology Co., Shanghai, China) at 37 °C in the dark for 20 min. Thereafter, the staining solution was removed and washed with PBS twice. Then, the morphology of treated cells was observed under a fluorescence microscope (XD-202, NOVEL, Nanjing, China).

### 5.6. Annexin V-FITC/Propidium Iodide (PI) Assay

Annexin V-FITC and propidium iodide (PI) staining were performed as previously described with some modifications [48]. Briefly, GES-1 cells were treated with various concentrations of MPN for 16 h. Then, the cells were harvested by centrifugation at 2000 rpm for 5 min (D2012, SCILOGEX, Pittsburgh, PA, USA), washed with PBS, and resuspended in 500 μL of binding buffer. Thereafter, 5 μL of Annexin V-FITC and PI were added and incubated in the dark at room temperature for 15 min. The stained cells were analyzed by using a flow cytometry (AccuriTM-C6, BD Biosciences, San Diego, CA, USA).

### 5.7. Cell Cycle Analysis

The PI staining was performed to analyze the changes that took place in the cell cycle upon MPN treatment. As described above, 1 × 10^4^ cells per well in a 96-well plate were seeded and treated with MPN for 16 h. After that, cells were transferred to 70% cold ethanol, then collected at 2000 rpm (D2012, SCILOGEX, Pittsburgh, PA, USA) for 5 min, and resuspended in PBS. Cells, then, were centrifuged again at 2000 rpm for 5 min and treated with 100 μL of RNAse in a water bath at 37 °C for 30 min. Lastly, 400 μL of PI (50 μg/mL) was added and incubated at 4 °C in dark for 30 min. The PI fluorescence was read on a flow cytometry at 488 nm.

### 5.8. Detection of Mitochondrial Membrane Potential

The mitochondrial potential (ΔΨm) was measured by using JC-1 mitochondrial membrane potential detection kit (BA1450, Enogene Biotechnology Co., Nanjing, China) according to the manufacturer’s instructions. In brief, GES-1 cells were treated with various concentrations of MPN at 37 °C for 16 h. Then, the treated cells were washed with PBS twice, suspended in 500 μL JC-1 dye, and incubated for 15 min. After that, samples were centrifuged at 2000 rpm for 5 min, then resuspended in 500 μL staining binding solution. Finally, the red and green fluorescence was observed with flow cytometry at 488 nm.

### 5.9. Western Blotting Analysis

The cell samples were separated by sodium dodecyl sulfate-polyacrylamide gel electrophoresis (SDS-PAGE). Subsequently, the proteins were transferred onto a polyvinylidene fluoride (PVDF, Merck Millipore, Billerica, MA, USA) membrane and incubated overnight at 4 °C with the following primary antibodies: Caspase-8 antibody (E18-5267, Enogene Biotechnology Co., Nanjing, China), Bax antibody (E18-0083, Enogene Biotechnology Co., Nanjing, China), and β-Tublin antibody (E12-043, Enogene Biotechnology Co., Nanjing, China). Next, the PVDF membrane was washed with TBST three times, and incubated for 2 h with the following secondary antibodies: HRP-labeled goat anti-rabbit secondary antibody (Enogene Biotechnology Co., Nanjing, China), HRP-labeled goat anti-mouse secondary antibody (Enogene Biotechnology Co., Nanjing, China). Finally, immunoreactivity was detected by using an ECL Plus chemiluminescence detection kit (P0018A, Beyotime Co., Shanghai, China) and a Fluor Chem M system (Protein Simple, Santa Clara, CA, USA). The gray value of each band was calculated by using Photoshop software.

### 5.10. Statistical Analysis

The measurement data were expressed as mean ± standard deviation. The results were subjected to analysis of variance (ANOVA) using the Statistical Package for the Social Sciences (SPSS Inc., Chicago, IL, USA). GraphPad Prism Version 8.0 (GraphPad Software, San Diego, CA, USA) for Windows was used to calculate the IC_50_ value. Significant differences between sample means were determined by using student’s *t*-test at *p* < 0.05.

## Figures and Tables

**Table 1:** The orthogonal test results with L_9_(3^4^).

Test No.	Factors				Extraction Amount of 4′-O-methylpyridoxine (MPN) (μg/mg)
	A: Temperature (°C)	B: Solid-to-Liquid Ratio (m/v)	C: Time(min)	Blank	
1	1(40)	1(1:5)	1(60)	1	1.870
2	1	2(1:10)	2(80)	2	2.007
3	1	3(1:15)	3(100)	3	1.777
4	2(50)	1	2	3	1.928
5	2	2	3	1	1.913
6	2	3	1	2	1.496
7	3(60)	1	3	2	1.455
8	3	2	1	3	1.376
9	3	3	2	1	1.123

**Table 2:** Analysis of variance.

Source of Variation	Sum of Squares	Degree of Freedom	F Value	F_0.05(2.2)_ = 19	F_0.01(2.2)_ = 99
A: Temperature	0.545	2	109.000	*	* *
B: Solid-to-liquid ratio	0.172	2	34.400	*	
C: Time	0.030	2	6.000		
Blank	0.005	2	1.000		
Error	0.01	2			

“**” means the difference is significant at the 0.01 level, while “*” means the difference is significant at the 0.05 level.

## Data Availability

No new data were created or analyzed in this study. Data sharing is not applicable to this article.

## Supplementary Materials

The following are available online at https://www.mdpi.com/2072-6651/13/2/95/s1, Figure S1: Effect of temperature on extraction amount of MPN, Figure S2: Effect of extraction time on extraction amount of MPN, Figure S3: Effect of solid–liquid ratio on extraction amount of MPN; Figure S4: Inhibitory effect of MPN with different concentration on cells under different treatment time; Table S1: Calibration curves, R2, linear range, LOD, and LOQ for MPN, Table S2: Precision of the determination methods for MPN. The values were expressed as mean ± SE (n = 6), Table S3: Accuracy of the determination methods for MPN. The values were expressed as mean ± SE (n = 3); Table S4: The percentages of GES-1 cells in different quadrants of scatterplot from flow cytometry; Table S5: Effect of MPN on cell-cycle distribution of GES-1 cells; Table S6: The change of fluorescence of MPN-treated GES-1 cells stained with JC-1; Table S7: The correlation coefficients of the eight indicators; Table S8: The IC50 values of MPN at different treatment time.

## References

[1] Zhang W., Zou M., Wu R., Jiang H., Cao F., Su E. (2019). Insight into the transformation of 4′-O-methylpyridoxine and 4′-O-methylpyridoxine-5′-glucoside in Ginkgo biloba seeds undergoing the heat treatment. Ind. Crop. Prod..

[2] Gong H., Wu C., Kou X.-H., Fan G.-J., Li T.-T., Wang J.-H., Wang T. (2019). Comparison study of 4′-O-methylpyridoxine analogues in Ginkgo biloba seeds from different regions of China. Ind. Crop. Prod..

[3] Wada K., Ishigaki S., Ueda K., Sakata M., Haga M. (1985). An antivitamin B6, 4′-methoxypyridoxine, from the seed of *Ginkgo biloba* L.. Chem. Pharm. Bull..

[4] Scott P.M., Lau B.P.-Y., A Lawrence G., A Lewis D. (2000). Analysis of Ginkgo biloba for the Presence of Ginkgotoxin and Ginkgotoxin 5′-Glucoside. J. AOAC Int..

[5] Leistner E., Drewke C. (2010). Ginkgo biloba and Ginkgotoxin. J. Nat. Prod..

[6] Arenz A., Klein M., Fiehe K., Groß J., Drewke C., Hemscheidt T., Leistner E. (1996). Occurrence of Neurotoxic 4′-O-Methylpyridoxine in Ginkgo biloba Leaves, Ginkgo Medications and Japanese Ginkgo Food. Planta Med..

[7] Hori Y., Fujisawa M., Shimada K., Oda A., Katsuyama S., Wada K. (2004). Rapid Analysis of 4-O-Methylpyridoxine in the Serum of Patients with Ginkgo Biloba Seed Poisoning by Ion-Pair High-Performance Liquid Chromatography. Biol. Pharm. Bull..

[8] Lawrence G.A., Scott P.M. (2005). Improved extraction of ginkgotoxin (4′-O-methylpyridoxine) from Ginkgo biloba products. J. Aoac Int..

[9] Yoshimura T., Udaka N., Morita J., Zhang J.Y., Sasaki K., Kobayashi D., Wada K., Hori Y. (2006). High performance liquid chroma-tographic determination of ginkgotoxin and ginkgotoxin-5′-glucoside in Ginkgo biloba seeds. J. Liquid Chromatogr. Relat. Technol..

[10] Kobayashi D., Yoshimura T., Johno A., Sasaki K., Wada K. (2011). Toxicity of 4′-O-methylpyridoxine-5′-glucoside in Ginkgo biloba seeds. Food Chem..

[11] Havaux M., Ksas B., Szewczyk A., Rumeau D., Franck F., Caffarri S., Triantaphylidès C. (2009). Vitamin B6 deficient plants display increased sensitivity to high light and photo-oxidative stress. BMC Plant Biol..

[12] Mei N., Guo X., Ren Z., Kobayashi D., Wada K., Guo L. (2017). Review of Ginkgo biloba-induced toxicity, from experimental studies to human case reports. J. Environ. Sci. Health Part C.

[13] Miwa H., Iijima M., Tanaka S., Mizuno Y. (2001). Generalized convulsions after consuming a large amount of Gingko nuts. Epilepsia.

[14] Kajiyama Y., Fujii K., Takeuchi H., Manabe Y. (2002). Ginkgo Seed Poisoning. Pediatrics.

[15] Kerr J.F.R., Wyllie A.H., Currie A.R. (1972). Apoptosis: A basic biological phenomenon with wide-ranging implication in tissue kinetics. Br. J. Cancer.

[16] Baar M.P., Brandt R.M.C., Putavet D.A., Klein J.D.D., Derks K.W.J., Bourgeois B.R.M., Stryeck S., Rijksen Y., Van Willigenburg H., Feijtel D.A. (2017). Targeted Apoptosis of Senescent Cells Restores Tissue Homeostasis in Response to Chemotoxicity and Aging. Cell.

[17] Candeias E., Sebastiao I., Cardoso S., Carvalho C., Santos M.S., Oliveira C.R., Moreira P.I., Duarte A.I. (2018). Brain GLP-1/IGF-1 Sig-naling and Autophagy Mediate Exendin-4 Protection Against Apoptosis in Type 2 Diabetic Rats. Mol. Neurobiol..

[18] Pfeffer C.M., Singh A.T.K. (2018). Apoptosis: A Target for Anticancer Therapy. Int. J. Mol. Sci..

[19] Pena-Blanco A., Garcia-Saez A.J. (2018). Bax, Bak and beyond—Mitochondrial performance in apoptosis. Febs J..

[20] Levine S., Saltzman A. (2002). Pyridoxine (vitamin B6) toxicity: Enhancement by uremia in rats. Food Chem. Toxicol..

[21] Schaumburg H., Kaplan J., Windebank A., Vick N., Rasmus S., Pleasure D., Brown M.J. (1983). Sensory neuropathy from pyridoxine abuse. A new megavitamin syndrome. N. Engl. J. Med..

[22] Fiehe K., Arenz A., Drewke C., Hemscheidt T., Williamson R.T., Leistner E. (2000). Biosynthesis of 4′-O-methylpyridoxine (Gink-gotoxin) from primary precursors. J. Nat. Prod..

[23] Galluzzi L., Vitale I., Senovilla L., Olaussen K.A., Pinna G., Eisenberg T., Goubar A., Martins I., Michels J., Kratassiouk G. (2012). Prognostic Impact of Vitamin B6 Metabolism in Lung Cancer. Cell Rep..

[24] Hardwick J.M., Soane L. (2013). Multiple functions of BCL-2 family proteins. Cold Spring Harb. Perspect. Biol..

[25] Van Opdenbosch N., Lamkanfi M. (2019). Caspases in cell death, inflammation, and disease. Immunity.

[26] Fan T.-J., Han L.-H., Cong R.-S., Liang J. (2005). Caspase Family Proteases and Apoptosis. Acta Biochim. Biophys. Sin..

[27] Akl H., Vervloessem T., Kiviluoto S., Bittremieux M., Parys J.B., De Smedt H., Bultynck G. (2014). A dual role for the anti-apoptotic Bcl-2 protein in cancer: Mitochondria versus endoplasmic reticulum. Biochim. Biophys. Acta BBA Bioenerg..

[28] Birkinshaw R.W., Czabotar P.E. (2017). The BCL-2 family of proteins and mitochondrial outer membrane permeabilisation. Semin. Cell Dev. Biol..

[29] De Giorgi F., Lartigue L., Bauer M.K., Schubert A., Grimm S., Hanson G.T., Remington S.J., Youle R.J., Ichas F. (2002). The perme ability transition pore signals apoptosis by directing Bax translocation and multimerization. FASEB J..

[30] Darendelioglu E., Aykutoglu G., Tartik M., Baydas G. (2016). Turkish propolis protects human endothelial cells In Vitro from ho-mocysteine-induced apoptosis. Acta Histochem..

[31] Ma L., Wang X., Li W., Qu F., Liu Y., Lu J.-C., Su G., Zhao Y. (2019). Conjugation of Ginsenoside with Dietary Amino Acids: A Promising Strategy to Suppress Cell Proliferation and Induce Apoptosis in Activated Hepatic Stellate Cells. J. Agric. Food Chem..

[32] Sanderson T.H., Reynolds C.A., Kumar R., Przyklenk K., Hüttemann M. (2013). Molecular Mechanisms of Ischemia–Reperfusion Injury in Brain: Pivotal Role of the Mitochondrial Membrane Potential in Reactive Oxygen Species Generation. Mol. Neurobiol..

[33] Bayomy N.A., Abdelaziz E.Z., Said M.A., Badawi M.S., El-Bakary R.H. (2016). Effect of pycnogenol and spirulina on vancomy-cin-induced renal cortical oxidative stress, apoptosis, and autophagy in adult male albino rat. Can. J. Physiol. Pharmacol..

[34] Li S.-Z., Ren J., Fei J., Zhang X., Du R.-L. (2018). Cordycepin induces Bax-dependent apoptosis in colorectal cancer cells. Mol. Med. Rep..

[35] Fischer U.M., Janicke R.U., Schulze-Osthoff K. (2003). Many cuts to ruin: A comprehensive update of caspase substrates. Cell Death Differ..

[36] Vrolijk M.F., Opperhuizen A., Jansen E.H.J.M., Hageman G.J., Bast A., Haenen G.R.M.M. (2017). The vitamin B6 paradox: Supplemen-tation with high concentrations of pyridoxine leads to decreased vitamin B6 function. Toxicol. Vitro.

[37] Ueland P.M., McCann A., Midttun Ø., Ulvik A. (2017). Inflammation, vitamin B6 and related pathways. Mol. Asp. Med..

[38] Škorňa P., Rimarčík J., Poliak P., Lukes V., Klein E. (2016). Thermodynamic study of vitamin B6 antioxidant potential. Comput. Theor. Chem..

[39] Fitzpatrick T.B., Amrhein N., Kappes B., Macheroux P., Tews I., Raschle T. (2007). Two independent routes of de novo vitamin B6 biosynthesis: Not that different after all. Biochem. J..

[40] Lebiedzińska A., Marszałł M.L., Grembecka M., Czaja J., Szefer P., Kuta J., Garabato B.D., Kozlowski P.M. (2017). Detection of vitamin B6 in grain products: Experimental and computational studies. Food Anal. Methods.

[41] Schmidt A., Schreiner M., Mayer H.K. (2017). Rapid determination of the various native forms of vitamin B6 and B2 in cow’s milk using ultra-high performance liquid chromatography. J. Chromatogr. A.

[42] Kästner U., Hallmen C., Wiese M., Leistner E., Drewke C. (2007). The human pyridoxal kinase, a plausible target for ginkgotoxin fromGinkgo biloba. FEBS J..

[43] Fitzpatrick T.B., Basset G.J., Borel P., Carrari F., DellaPenna D., Fraser P.D., Hellmann H., Osorio S., Rothan C., Valpuesta V. (2012). Vitamin Deficiencies in Humans: Can Plant Science Help?. Plant Cell.

[44] Kobayashi D., Yoshimura T., Johno A., Ishikawa M., Sasaki K., Wada K. (2015). Decrease in pyridoxal-5′-phosphate concentration and increase in pyridoxal concentration in rat plasma by 4′-O-methylpyridoxine administration. Nutr. Res..

[45] Buss K., Drewke C., Lohmann S., Piwonska A., Leistner E. (2001). Properties and interaction of heterologously expressed glutamate decarboxylase isoenzymes GAD(65kDa) and GAD(67kDa) from human brain with ginkgotoxin and its 5′-phosphate. J. Med. Chem..

[46] Gong H., Wu C., Fan G.-J., Li T.-T., Wang J., Wang T. (2018). Determination and Comparison of 4′-O-Methylpyridoxine Analogues in Ginkgo biloba Seeds at Different Growth Stages. J. Agric. Food Chem..

[47] Gong H., Wu C.-E., Kou X.-H., Fan G.-J., Li T.-T., Wang J.-H., Wang T. (2020). Determination of the native contents of 4′-O-methylpyridoxine and its glucoside in Ginkgo biloba Seeds. J. Food Meas. Charact..

[48] Madeo F., Fröhlich E., Fröhlich K.-U. (1997). A Yeast Mutant Showing Diagnostic Markers of Early and Late Apoptosis. J. Cell Biol..

