# Supplementary material for: 4′-O-methylpyridoxine: Preparation from Ginkgo biloba Seeds and Cytotoxicity in GES-1 Cells
Source: Toxins (Basel). 2021 Jan 26;13(2):95. doi: 10.3390/toxins13020095 (PMC7912177; doi:10.3390/toxins13020095)
Supplement: Supplementary file 1 [file toxins-13-00095-s001.pdf]

Supplementary Materials: 4'-*O*-Methylpyridoxine: Preparation from *Ginkgo Biloba* Seeds and Cytotoxicity in GES-1 Cells

Jin-Peng Zhu [1], Hao Gong [2], Cai-E Wu [1,3,4*], Gong-Jian Fan [1,3,4], Ting-Ting Li [1,3,4] and Jia-Hong Wang [1,3,4]

1. Methodology validation of HPLC methods

The methodology validation for the determination of the MPN in *G .biloba* seeds were investigated. The equation of the regression line, correlation coefficient (R^2), linear range, limit of detection (LOD), limit of quantification (LOQ), precious test, and recovery for each compound were presented in Table S1.

Standard curves: The standards of the MPN were diluted with distilled water (adjusted to pH 2.5 by using phosphoric acid) to obtain solutions at concentrations of 0.01, 0.05, 0.1, 0.5, 1.0, and 1.5 mg/mL. The samples were injected into the HPLC system under chromatographic conditions, and chromatograms were recorded at an excitation wavelength of 295 nm and an emission wavelength of 395 nm. The calibration curves were constructed by plotting the peak area versus the concentration, and the regression equations were calculated for each analysis.

LOD and LOQ: LOD and LOQ were determined at a signal to-noise ratio (S/N) of 3 and 10, respectively. LOD and LOQ were experimentally verified by five injections of MPN at the LOD and LOQ concentration range.

Precision: MPN was verified by within-day variations of replicate determinations. The standard solutions (0.5, 1.0, and 10.0 μg/mL) of MPN was injected six times, and the measured concentration was recorded. The results of precision test were reported in terms of relative standard deviation (RSD) in Table S2.

Accuracy: the known amounts of MPN standard solutions (5, 50, and 100 μg/mL) were added into the extraction solutions of freeze-dried *G. biloba* seed powder. The recovery rate was calculated as the ratio between the increased amounts and the added amounts of MPN, and the RSD was also obtained in the three replicated (Table S3). RSD at the range of ±5% showed the methods were deemed to be accurate.

2. Effect of extraction temperature on extraction amount of MPN

MPN will be converted into MPNG, when the temperature at a high level, which will reduce the extraction amount of MPN. The effect of temperature on the dissolution of MPN from *G. biloba* seeds powder is shown in Fig. S1. With the increase of temperature, the extraction amount of MPN increased slowly and then decreased sharply. In the temperature range of 30°C to 50°C, the extraction amount increased slowly and reached the maximum value of 0.47 μg/mg at 50°C. When the temperature was higher than 50°C, the extraction amount of MPN decreased rapidly and reached the lowest value of 0.24 μg/mg at 80°C.

Therefore, the extraction temperature of 50°C was selected as the temperature for further optimization of extraction conditions.

3. Effect of extraction time on extraction amount of MPN

The extraction time has an effect on the extraction amount of MPN from *G. biloba* seeds powder. The influence of extraction time on the extraction amount of MPN is shown in Fig. S2. With the extension of extraction time, the extraction amount of MPN increased slowly. When the extraction time exceeded 100 min, the extraction amount of MPN decreased as a whole.

With the time increased, water and *G. biloba* seeds powder contact more and more fully, which is conducive to the dissolution of MPN. When time is 100 min, the extraction amount of MPN reaches the maximum value of 2.36 μg/mg. Heat production in the shaking process may lead to an increase in the overall temperature of the extraction solution, which is conducive to the conversion of MPN to MPNG, which results in the overall low extraction amount of MPN when the extraction time is 120min-180min. Therefore, the extraction time of 100 min can be selected as the time range for further optimization.

4. Effect of solid-liquid ratio on extraction amount of MPN

The ratio of solid to liquid can also affect the extraction amount of MPN. The effect of the amount of water added on the extraction amount of MPN in *G. biloba* seeds powder is shown in Fig. S3. When the ratio of solid-liquid was 1:5 to 1:20, the change was obvious, which first increased and then decreased. When the ratio of solid-liquid was 1:10, the extraction amount of MPN reached the maximum value of 1.32 μg/mg. The extraction of MPN reached the minimum value of 0.72 μg/mg at 1:40. Therefore, the solid-liquid ratio of about 1:10 was selected as the range of material-liquid ratio for further optimization of extraction conditions.

Table S1. Calibration curves, R^2, linear range, LOD, and LOQ for MPN.

	**Calibration curves**	**R^2**	**Line range(μg/mL)**	**LOD (μg/mL)**	LOQ (μg/mL)
**MPN**	**$y = 381.39x - 175944$**	**0.9979**	**0.015-10**	**0.031**	0.135

Table S2. Precision of the determination methods for MPN. The values was expressed as mean ± SE (n=6).

	**Estimated value (μg/mL)**			**RSD (%)**
	**5 μg/mL**	**50 μg/mL**	**100 μg/mL**	
MPN	4.99±0.21	50.31±0.73	100.25±0.92	2.19

Table S3. Accuracy of the determination methods for MPN. The values was expressed as mean ± SE (n=3).

	**Recovery rate (%)**			**RSD (%)**
	**5 μg/mL**	**50 μg/mL**	**100 μg/mL**	
MPN	98.89±3.21	100.33±0.73	100.68±0.92	1.62

Table S4. The percentages of GES-1 cells in different quadrants of scatterplot from flow cytometry.

**Group**	**UL (%)**	**LL (%)**	**UR (%)**	**LR (%)**	**Apoptotic rate (%)**
Blank	2.70 ± 0.71a	92.90 ± 0.20a	2.45 ± 0.55c	1.97 ± 0.35c	4.41 ± 0.895c
5μM	2.32 ± 1.25ab	90.80 ± 2.10a	3.73 ± 2.06c	3.11 ± 1.29c	6.84 ± 3.35c
50μM	0.871 ± 0.26b	84.70 ± 1.90b	6.93 ± 1.35b	7.53 ± 0.82b	14.46 ± 2.17b
100μM	2.48 ± 0.80ab	53.75 ± 0.95c	17.66 ± 2.26a	26.15 ± 0.45a	43.80 ± 1.81a

Note: UL: Necrotic cells; LL: Living cells; UR: Late apoptotic cells; LR: Early apoptotic cells. Different lowercase letters indicate the difference of different administered MPN concentrations at $p < 0.05$.

Table S5. Effect of MPN on cell-cycle distribution of GES-1 cells.

**Group**	**G1/ G0 (%)**	**S (%)**	**G2/M (%)**
Blank	51.98 ± 0.67b	44.43 ± 0.49a	3.59 ± 0.17c
5μM	55.54 ± 0.65b	41.99 ± 0.25a	2.48 ± 0.90c
50μM	55.24 ± 2.17b	39.65 ± 3.99a	5.12 ± 1.82b
100μM	63.39 ± 7.62a	26.79 ± 7.46b	9.84 ± 0.16a

Different lowercase letters indicate the difference of different administered MPN concentrations at $p < 0.05$.

Table S6. The change of fluorescence of MPN-treated GES-1 cells stained with JC-1.

**Group**	**Green fluorescence intensity**
Blank	3.76 ± 0.27c
5μM	5.18 ± 0.83c
50μM	28.45 ± 1.15b
100μM	30.35 ± 1.35a

Different letters indicate the difference of different administered MPN concentrations at $p < 0.05$.

Table S7. The correlation coefficients of the eight indicators.

	Inhibition rate	Apoptotic rate	Cells in G1/G0	Cells in S	Cells in G2/M	Mitochondrial membrane depolarization	Caspase 8 expression	BAX expression
**Inhibition rate**	1	0.994**	0.949	−0.989*	0.982*	0.850	0.969*	0.985*
**Apoptotic rate**		1	0.966*	−0.997**	0.978*	0.785	0.984*	0.995*
**G1/G0**			1	−0.982*	0.891	0.710	0.996**	0.940
**S**				1	−0.960*	−0.773	−0.994**	−0.986*
**G2/M**					1	0.816	0.926	0.989*
**Green fluorescence intensity**						1	0.726	0.759
**Caspase 8**							1	0.967*
**Bax**								1

"**" means the correlation is significant at the 0.01 level, while "*" means the correlation is significant at the 0.05 level.

Table S8. The IC_{50} values of MPN at different treatment time.

**Time (h)**	**6**	**10**	**16**	**24**	**48**	**72**
IC_{50} (μM)	242.80	245.40	132.70	151.80	359.90	389.90

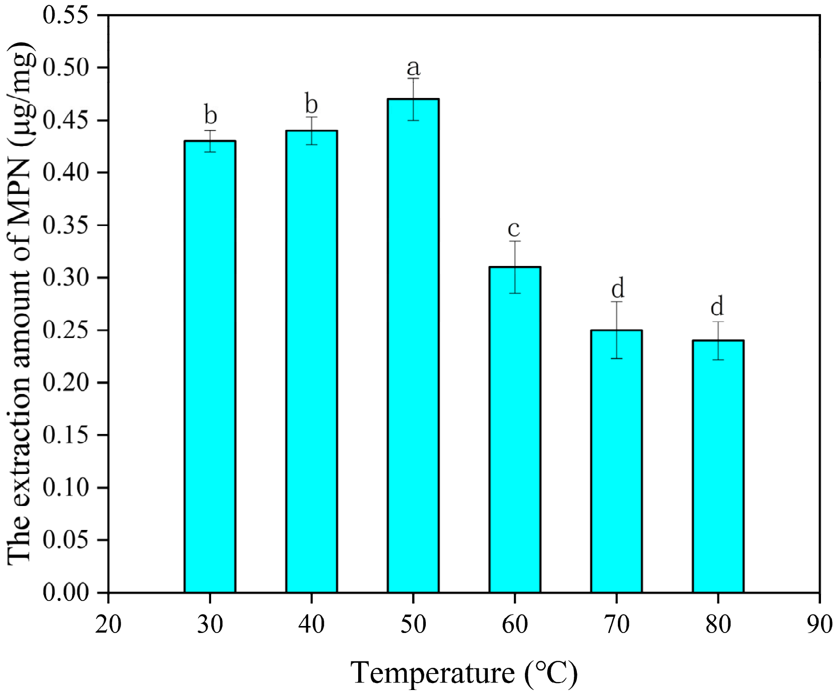

Figure S1. Effect of temperature on extraction amount of MPN.

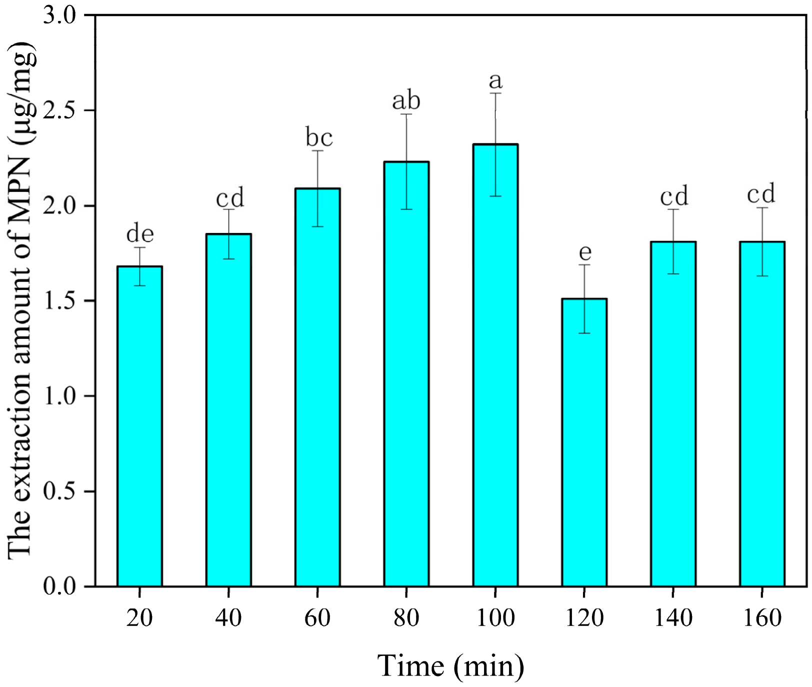

Figure S2. Effect of extraction time on extraction amount of

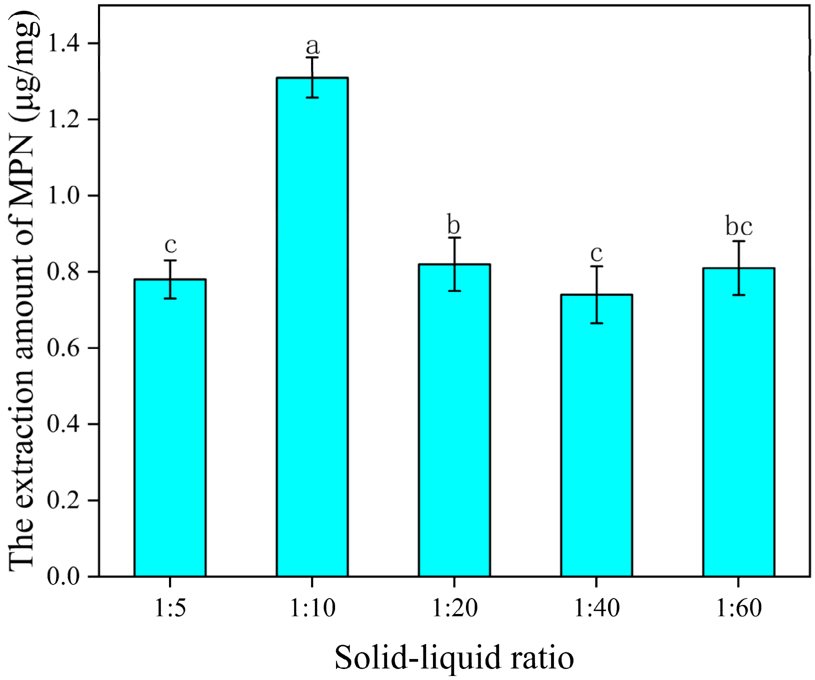

MPN.

Figure S3. Effect of solid-liquid ratio on extraction amount of MPN.

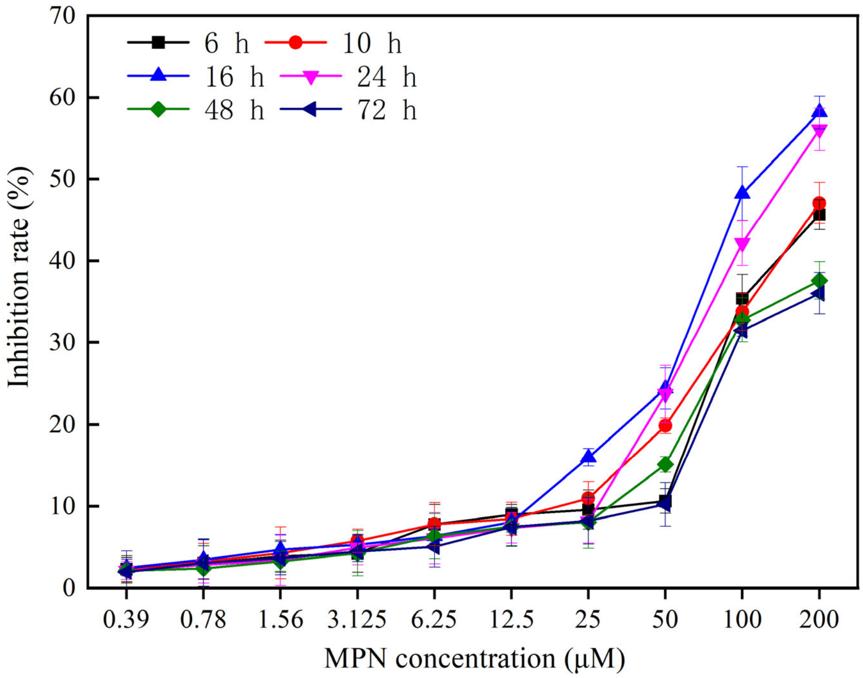

Figure S4. Inhibitory effect of MPN with different concentration on cells under different treatment time.
